# Transanal total mesorectal excision (TaTME) in rectal cancer treatment within an expert center

**DOI:** 10.1038/s41598-023-44247-8

**Published:** 2023-10-10

**Authors:** Jacek Piątkowski, Mateusz Jagielski, Jacek Szeliga, Mariusz Nowak, Marek Jackowski

**Affiliations:** Department of General, Gastroenterological and Oncological Surgery, Collegium Medicum Nicolaus Copernicus University, 53-59 Św. Józefa St, 87-100 Toruń, Poland

**Keywords:** Outcomes research, Colorectal cancer

## Abstract

The objective of this study was to evaluate treatment outcomes in patients who underwent the TaTME procedure for cancer of the middle and low rectum in an expert center. Prospective analysis of the outcomes of all consecutive patients treated using the TaTME technique for cancer of the middle and distal rectum at the our medical center between March 1, 2015, and March 31, 2022. A total of 128 patients (34 women, 94 men; mean age 66.01 [38–85] years) with cancer of the middle and distal rectum qualified for TaTME. TaTME procedures were performed in 127/128 (99.22%) patients. Complications of surgery were observed in 22/127 (17.32%) patients. Negative proximal and distal margins were confirmed in all 127 patients. Complete (R0) resection of the mesorectum was confirmed in 125/127 (98.43%) and nearly complete (R1) resection was confirmed in 2/127 (1.57%) patients. The average follow-up period was 795 days (296–1522) days. Local recurrence was detected during the follow-up period in 2/127 (1.57%) patients. This study showed that the TaTME procedure is an effective and safe method for the minimally invasive treatment of middle and low rectal cancers, particularly within an expert center setting.

## Introduction

Surgical resection, frequently combined with preoperative radiotherapy or radiochemotherapy, is the recommended therapeutic approach in the management of patients with rectal cancer^1–3^. Oncological treatment in form of radiotherapy or chemoradiotherapy plays a crucial role in many rectal cancers: firstly—by decreasing rates of local recurrence, secondly—by making some rectal cancers resectable by decreasing size of the tumor, and thirdly—by limiting or even avoiding surgical treatment^1–3^. Although combined multidisciplinary management of rectal cancer improves oncological outcomes^1–3^, total mesorectal excision (TME) remains the gold standard for the treatment of locally advanced rectal cancer. Regardless of the surgical technique, the TME approach aims to reduce local recurrence, thereby increasing survival in rectal cancer patients, and is an important prognostic factor^4–6^.

Surgical procedures in patients with rectal cancer are technically difficult owing to anatomical conditions and remain a challenge even at expert centers^1–3,7–9^. In recent years, several minimally invasive techniques have been proposed for abdominal surgery, which, compared to conventional surgical treatment, reduce hospitalization time and improve patient outcomes^10–12^. Minimally invasive techniques have also been developed in the field of oncological surgery^7–9^. Laparoscopic TME (LaTME) is associated with better visualization of the pelvic cavity than compared to open surgery^13–15^. However, LaTME is associated with significant technical limitations, particularly in low rectal tumors, owing to the narrowness and depth of the surgical field, that is, the pelvis, making it difficult to obtain good oncological purity margins^13–15^. Minimally invasive surgical techniques for the treatment of low rectal cancer require further research to evaluate their efficacy and safety.

In addition to the short- and long-term oncological outcomes, the quality of life after surgery is an additional factor to be considered in the management of patients with rectal cancer. In low rectal tumors, preservation of sphincter function while obtaining clean resection margins remains an important issue. All the above criteria appear to be met by a technique referred to as transanal TME (TaTME), first described in the literature in 2010^16^. Later publications from the same research center^17,18^ reported studies conducted on a larger group of patients and seemed to confirm the effectiveness and safety of this novel surgical technique.

Despite the ongoing development of surgical techniques and various oncological treatments, low rectal cancer remains a challenge for both surgeons and oncologists, creating multidisciplinary teams. The objective of this study was to evaluate treatment outcomes in patients who underwent the TaTME procedure for cancer of the middle and low rectum. This paper presents an attempt to optimize the surgical treatment of rectal cancer within an expert center where procedures using advanced laparoscopic techniques are performed on a daily basis^19^.

## Methods

In this study, we prospectively analyzed the outcomes of all consecutive patients treated using the TaTME technique for cancer of the middle and distal rectum at the Department of General, Gastroenterological and Oncological Surgery of the Ludwik Rydygier Medical College in Bydgoszcz, Nicolaus Copernicus University in Toruń between March 1, 2015, and March 31, 2022.

The study was approved by the Ethics Committee of Collegium Medicum Nicolaus Copernicus University (institutional review board) and proceeded in line with the tenets set by the Declaration of Helsinki. All patients gave their informed consent for the procedures.

All patients were reviewed for surgical treatment at case conferences held within oncological teams. Neoadjuvant treatment was administered before surgery in most of patients. Patients qualified for TaTME if the rectal tumor was located within 50 mm from the pectinate line. TaTME was also performed when the tumor was located 50–100 mm away from the pectinate line if certain anatomical conditions (obesity with BMI over 35, male sex, and narrow pelvis) were met. In all other cases, patients with tumors in the middle rectum underwent LaTME.

### The TaTME procedure

All patients underwent surgery under general anesthesia and endotracheal intubation in the Lloyd-Davis position. The main surgeons involved in all the surgical procedures were Dr. Jacek Piątkowski and Prof. Marek Jackowski.

Surgeries were initiated by pneumoperitoneum created by the abdominal surgical team using a Veress needle inserted at the right edge of the umbilicus or, in exceptional situations, at Palmer’s point. A 10-mm optical trocar was inserted at the right edge of the umbilicus. The remaining three trocars were inserted under visual guidance on the right side of the abdomen at the lateral edge of the rectus sheath. The patient was positioned to facilitate visualization of the mesentery within the left half of the colon, along with the inferior mesenteric vein. After clipping, the vein was transected into the lower edge of the pancreas, and the left half of the colon was mobilized by extramedial dissection along Toldt's fascia. The splenic flexure was then released in a conventional manner. Since December 2016, splenic flexure release has been a procedural standard; before, the flexure was released only when excessive tension was expected within the anastomosis. The inferior mesenteric artery was dissected, clipped, and transected above the ostium of the left colic artery, as high ligation of the mesenteric artery is a typical procedure performed at our center. Following mobilization of the sigmoid colon, the peritoneum was incised within the pelvis minor and the rectum was released from the sides.

**Figure 1:**
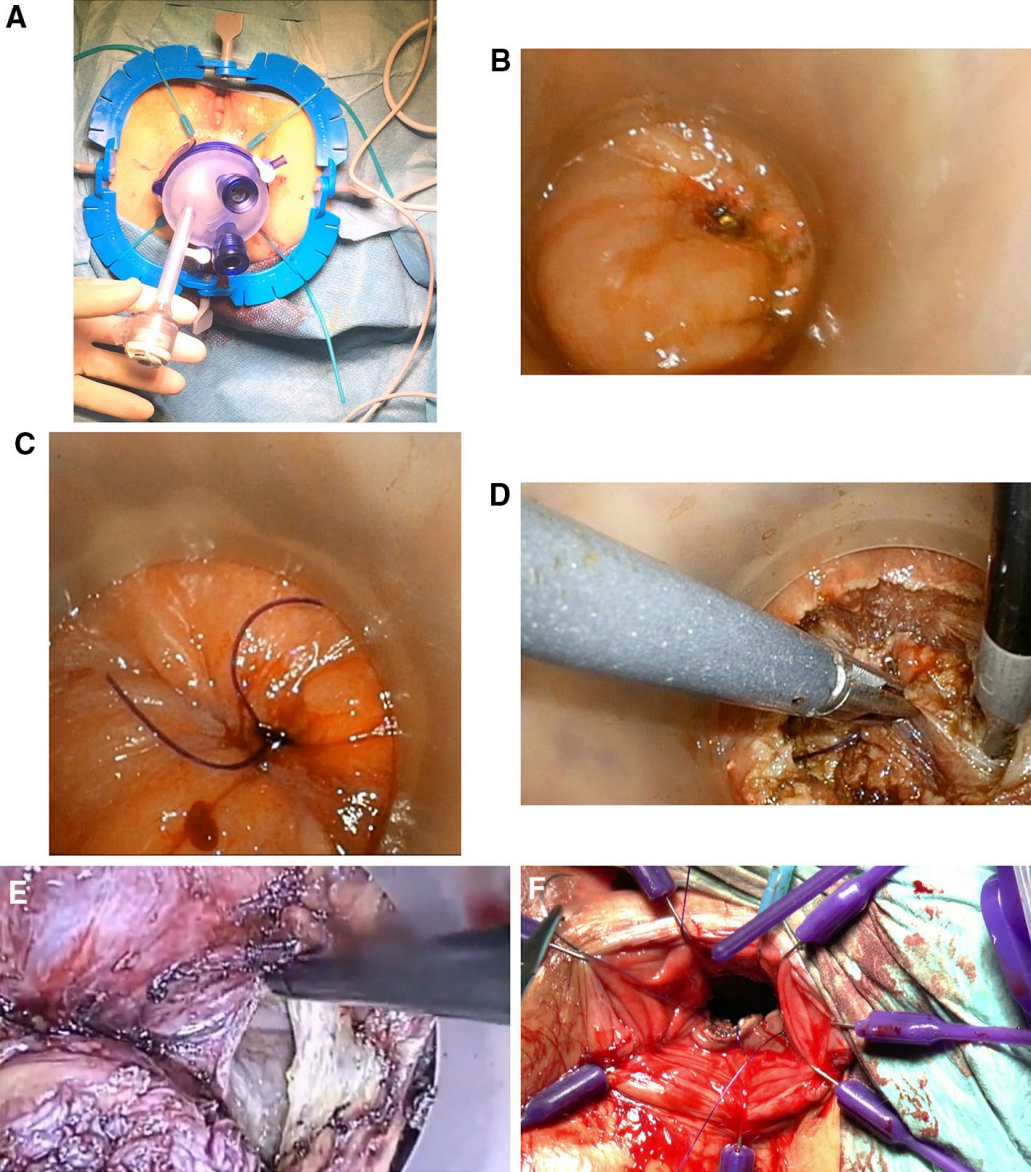
(**a**–**f**) TaTME procedure (access from the anal side). After introducing GelPort system into the rectum (**a**), the rectal lumen was closed approximately 10 mm below the tumor using a purse-string suture (**b**, **c**). Then the rectum was dissected in a circular manner by means of coagulation, proximally from the purse string, revealing presacral fascia (**d**, **e**). After the rectal excision, rectum with tumor was removed from the rectal stump (**f**).

Simultaneously, a second team of surgeons established access from the anal side (Fig. 1a–f). This was achieved by using the GelPort system. The rectal lumen was closed approximately 10 mm below the tumor using a purse-string suture, and octenidine solution was used to flush the rectal stump. A hook was used to cut the rectum in a circular fashion, approximately 10 mm peripherally from the suture. The rectum was resected upward along with the mesentery and surrounding fatty tissue. Once the peritoneal recess was reached and transected, the two surgical fields were merged. The rectum along with the tumor was pulled out through the anus. To date, extraction of the rectum using a Pfannenstiel incision has been required only in one case due to mesenteric fattening and a narrow pelvis. The previous abdominal surgical team had prepared the resection boundary, and the tumor was resected along the intestinal margins. Normal blood supply was confirmed using indocyanine green, and the postoperative rectal bed was flushed with octenidine solution. Next, purse-string sutures were placed on the proximal stump of the colon and the rectal stump. A circular stapler head was inserted into the sigmoid colon, the rectal purse-string suture was pulled, and a circular stapler was inserted through the anus. The size of the stapler depends on the bowel diameter (usually 29–31 mm). An end-to-end coloanal anastomosis was achieved using a stapler, and the tightness of the anastomosis was verified from the rectal side. If no microleaks were visible, the anastomosis was considered tight. If microleaks were detected, the anastomosis was sealed with single PDS 3-0 sutures. Indocyanine green was used again to confirm the proper intestinal blood supply. Beginning in December 2016, a protective ileostomy was established within the right iliac fossa. Before, ileostomy was performed based on the surgeon’s judgment. After hemostasis was established and peritoneal toileting was performed, a Redon drain was left within the pelvis.

### Postoperative period

Pain management was continued throughout the postoperative period. The Redon drain was removed after the patient was mobilized on postoperative day 1. Oral feeding was resumed on day 1 after surgery. If no complications occurred in the postoperative period, the patients were discharged on postoperative day 3.

In cases where an increase in inflammatory markers were observed in laboratory blood tests, or when patients presented with clinical signs suggestive of anastomotic dehiscence, endoscopic examination of the lower gastrointestinal tract was performed to assess the tightness of the anastomosis. If features of anastomotic leaks were identified on endoscopic examination, the diagnostics were expanded to include a multiphase contrast-enhanced computed tomography (CECT) scan of the abdomen and pelvis with contrast enhancement. If an anastomotic leak is confirmed, clinically stable patients without signs of diffuse peritonitis or septic features qualify for transanal endoscopic drainage^20^. Patients with anastomotic leakage and signs of diffuse peritonitis were eligible for surgical treatment.

### Follow-up

Follow-up outpatient visits were held every 3–6 months for the first three years after surgery, and every 12 months thereafter. During these visits, patients underwent physical examinations and carcinoembryonic antigen (CEA) determination. Multiphasic CECT examinations of the abdominal cavity, contrast-enhanced magnetic resonance examinations of the pelvis, and colonoscopy were performed every 12 months after surgery for the following five years or earlier if cancer recurrence was suspected. The last follow-up visit was conducted on February 20, 2023.

### Statistical analysis

All statistical calculations were performed using STATISTICA v. 10.0 data analysis software (StatSoft, Inc., 2011). Quantitative variables were characterized using arithmetic means, standard deviations, minimum and maximum values (ranges), and 95% CIs (confidence intervals). Qualitative variables were summarized as counts and percentages.

## Results

### Patient characteristics

A total of 128 patients (34 women, 94 men; mean age 66.01 [38–85] years) with cancer of the middle and distal rectum qualified for TaTME (Table 1).

**Table 1:** Characteristics of the patients from study group.

Number of patients underwent TaTME procedure, n (%)	127 (99.22%)
Age, mean [range]	66.01 [38–85]
Sex, n, men (%)	94 (73.44%)
Indications to surgery, n (%)	
Rectal adenocarcinoma	126 (98.44%)
Extensive polypoid nongranular laterally spreading tumor (villous adenoma with high-grade dysplasia)	2 (1.56%)
Distance of tumor from the pectinate line, mean [range]	50.5 [10–100] mm
0–50 mm away from the pectinate line, n (%)	71 (55.47%)
> 50 mm from the pectinate line, n (%)	57 (44.53%)
Neoadjuvant therapy, n (%)	93 (72.66%)
Radiotherapy	74 (57.82%)
Radiochemotherapy	19 (14.84%)
Interval between the completion of neoadjuvant treatment to surgery, mean [range]	11.87 [5–61] days

Of this cohort, 126/128 (98.44%) patients were diagnosed with rectal adenocarcinoma, and the 2/128 (1.56%) patients were diagnosed with an extensive polypoid nongranular laterally spreading tumor (LST-NG) following unsuccessful attempts at preoperative endoscopic treatment (villous adenoma with high-grade dysplasia was identified on histopathological examination).

The tumors were located at an average distance of 50.5 (10–100) mm from the pectinate line. In 71/128 (55.47%) patients, the tumor was located in the distal rectum (0–50 mm away from the pectinate line). In the remaining 57/128 (44.53%) patients, the tumor was located in the middle rectum (> 50 mm from the pectinate line).

Neoadjuvant cancer therapy was used in 93/128 (72.66%) patients. Of which, radiotherapy was administered in 74/128 (57.82%) patients, whereas the remaining 19/128 (14.84%) patients received radiochemotherapy. The mean interval between the completion of neoadjuvant treatment to surgery was 11.87 (5–61) days.

### Perioperative outcomes

TaTME procedures were performed in 127/128 (99.22%) patients (Table 2). In one patient, conversion to laparotomy (Hartmann's procedure) was performed because of local advancement of the tumor, and neoplastic infiltration of the urinary bladder and abdominal wall was detected intraoperatively. This patient was removed from the total number of study group.

**Table 2:** Perioperative and oncological outcomes.

Perioperative outcomes	
Time of the TaTME procedure, mean [range]	169 [110–260] min
Intraoperative blood loss, mean [range]	200 [100–550] mL
Prophylactic loop ileostomy, n (%)	80 (62.99%)
Complications of surgical treatment, n (%)	22 (17.32%)
Intestinal anastomotic leakage	10 (7.87%)
Ileostomy stricture	4 (3.15%)
Surgical site infections	2 (1.58%)
Others	6 (4.73%)
Duration of hospital stay, mean [range]	7.26 [4–48] days
Oncological outcomes	
Adenocarcinoma, n (%)	126 (99.21%)
G1	24
G2	46
G3	56
Villous adenoma with high-grade dysplasia and advanced fibrosis, n (%)	1 (0.79%)
TNM classification	
T1	14
T2	26
T3	81
T4	5
N0	58
N1	41
N2	21
N3	6
M0	126
Negative proximal margins, n (%)	127 (100%)
Negative distal margins, n (%)	127 (100%)
The quality of the mesorectum—radial margin	
Complete resection (R0), n (%)	125 (98.43%)
Nearly complete resection (R1), n (%)	2 (1.57%)
Adjuvant chemotherapy, n (%)	79 (62.2%)
Local recurrence during the follow-up period, n (%)	2 (1.57%)
Generalization of the neoplastic process, n (%)	6 (4.72%)

The mean duration of the TaTME procedure was 169 min (range, 110–260 min). The mean intraoperative blood loss was 200 mL (range, 100–550 mL). Prophylactic loop ileostomy was performed in 80/127 (62.99%) patients (Table 3).

**Table 3:** Evaluation of patients from the study group depending on the presence of protective ileostomy.

	With protective loop ileostomy	Without protective loop ileostomy
Number of patients, n (%)	80 (62.99%)	47 (37.01%)
Age, mean, [range]	65.62 [38–82]	67.83 [45–85]
Sex, n, men (%)	57 (71.25%)	37 (78.72%)
Distance of tumor from the pectinate line, mean, [range]	41.5 [10–100] mm	56.5 [10–100] mm
0–50 mm away from the pectinate line, n (%)	53 (66.25%)	18 (38.3%)
> 50 mm from the pectinate line, n (%)	27 (33.75%)	29 (61.7%)
Interval between the completion of neoadjuvant treatment to surgery, mean [range]	10.63 [5–47] days	12.01 [7–61] days
Time of the TaTME procedure, mean [range]	145 [110–220] min	178 [130–260] min
Complications of surgical treatment, n (%)	12 (15%)	10 (21.28%)
Duration of hospital stay, mean [range]	6.72 [4–36] days	8.21 [7–48]

Complications of surgical treatment were observed in 22/127 (17.32%) patients (Table 4).

**Table 4:** Evaluation of complications depending on neoadjuvant chemoradiation.

Complications of surgical treatment	With neoadjuvant chemoradiation	Without neoadjuvant chemoradiation
Intestinal anastomotic leakage, n (%)	8/93 (8.6%)	2/35 (5.71%)
Ileostomy stricture, n (%)	3/93 (3.23%)	1/35 (2.86%)
Surgical site infections, n, (%)	2/93 (2.15%)	0
Others, n (%)	5/93 (5.38%)	1/35 (2.86%)

The most common complication was intestinal anastomotic leakage, which was detected in 10/127 (7.87%) patients. Endoscopic negative pressure therapy was used in 5 patients (3.93%)^20^. Another 5 (3.93%) patients underwent additional surgical treatment (Hartmann's procedure was performed in 2 patients).

Ileostomy strictures requiring surgical intervention were identified in 4/127 (3.15%) patients, and surgical site infections were identified in 2/127 (1.58%) patients.

Other less common complications included urethral injury (1 patient), buttock abscess (1 patient), urinary retention (1 patient), and pneumonia (1 patient).

One patient was diagnosed with aortic thrombosis with extensive ischemia of the abdominal organs on the third postoperative day.

The mean duration of hospital stay was 7.26 (4–48) days. On the third post operative day 70/127 (55.12%) patients left the hospital.

### Oncological outcomes

Postoperative specimens obtained from 126/127 (99.21%) patients had rectal adenocarcinoma (G1, 24 patients; G2, 46 patients; G3, 56 patients). In 1/127 (0.79%) patients, villous adenoma with high-grade dysplasia and advanced fibrosis was detected in histopathological examination. The TNM classification of the tumors within the patient group was as follows: T1, 14 patients; T2, 26 patients; T3, 81 patients; T4, 5 patients; N0, 58 patients; N1, 41 patients; N2, 21 patients; N3, 6 patients; and M0, 126 patients. In all 5 T4 cases the tumor infiltrated prostate gland, which means that it invaded the surrounding organs in the lower rectum.

Negative proximal and distal margins were confirmed in all 127 patients.

The quality of the mesorectum, that is, the circular (radial) margin, was scored using three grades (complete, nearly complete, and incomplete)^21^. Complete (R0) resection of the mesorectum was confirmed in 125/127 (98.43%), nearly complete (R1) resection of the mesorectum was confirmed in 2/127 (1.57%) patients with radial margins < 1 mm, and no *incomplete* resections were identified (2 patients with T4 stage).

A total of 79/127 (62.2%) patients were qualified for adjuvant chemotherapy.

### Long-term results

The average follow-up period was 795 days (296–1522) days.

Local recurrence was detected during the follow-up period in 2/127 (1.57%) patients.

Generalization of the neoplastic process was observed in 6/127 (4.72%) patients during the follow-up period (metastatic lesions within the lungs, two patients; liver, two patients; bones, one patient; and adrenal glands, one patient). Four of the 6 patients with generalized malignancy received palliative chemotherapy, while metastasectomy was performed in the remaining 2 patients.

During the follow-up 7 patients had been lost. Four patients with generalized malignancy passed away during palliative chemotherapy. Other three patients did not attend outpatient control visits.

## Discussion

To date, many studies have shown that minimally invasive techniques improve surgical outcomes in patients with gastrointestinal cancers^22–24^. Minimally invasive methods used in rectal cancer surgery include laparoscopic techniques, particularly LaTME, as performed from the abdominal access, and TaTME in which abdominal and transanal access are used simultaneously^13–15^. The distal location of rectal tumors is a limitation of LaTME while simultaneously being an indication for TaTME. Compared to LaTME, TaTME facilitates a more accurate determination of the distal resection margin in rectal cancer, and better visualization of the distal part of the rectum makes it possible to perform a thorough dissection of the pelvis minor, even in cases of deep, narrow pelvis in male patients or in obese patients^25,26^. As demonstrated in our study, TaTME facilitates the complete excision of rectal tumors along with the mesorectum while preserving anal sphincter function.

The robotic colorectal surgery was introduced in our medical center in 2023. Period of this study was between March 1, 2015, and March 31, 2022. That is why the results from robotic surgery could not be included in this study.

The basic premise of cancer surgical treatment is that complete resection of the tumor is performed with negative resection margins, that is, free of cancer cells. In the case of rectal tumors, this translates to oncological purity of the proximal and distal margins and, above all, the circumferential margin, which ensures R0 resection^4–7,9^. While obtaining a negative proximal margin during rectal tumor resection is usually not problematic, negative distal and circumferential margins are difficult to obtain, particularly in cases of low rectal tumors^4–7^. As mentioned above, an advantage of transanal access in the TaTME procedure consists in better visualization of the surgical field, which facilitates high-quality dissection and thus optimal oncological treatment outcomes in malignancies located within the middle and the lower third of the rectum^16–19^. Negative proximal and distal margins were obtained from all patients in the study group. Two patients (1.57%) had positive circumferential margins. In both cases, no tumor cells were found along the incision line; however, the radial margin was < 1 mm, indicating an R1 resection (nearly complete TME).

Two (1.57%) patients in our study group experienced local recurrence after TaTME surgery; this result is very good in relation to the literature data and confirms the effectiveness of TaTME in the pursuit of improved oncological outcomes in patients with rectal cancer. The patients with local recurrence (1.57%—2 patients) are the same patients who had positive circumferential margins (1.57%—2 patients) despite use of adjuvant chemotherapy. In a study by Deijen et al.^27^, the risk of local recurrence in patients who underwent TaTME was 4%. On the other hand, in a 2022 study by Lin et al.^28^, local cancer recurrence was observed in 6 (9.5%) patients operated on for low rectal tumors using TaTME as compared to 15 (23.8%) patients operated on using the LaTME technique. As confirmed by these data, compared to the procedure carried out using abdominal access (LaTME), where complete excision of the mesorectum from the abdomen toward the pelvic floor is more difficult, particularly in patients with low rectal cancer, the TaTME technique, which uses transanal access, ensures better visibility of the mesorectum, facilitates preservation of adequate oncological margins, and thus reduces the risk of local cancer recurrence^27–29^.

The TaTME procedure is increasingly used in the treatment of rectal tumors despite the lack of standardized indications, training programs associated with the learning curve, or clearly defined efficacy and safety of this surgical technique. In our study, we demonstrated that TaTME is a novel, minimally invasive surgical procedure with the primary goal not only to improve oncological outcomes but also to improve the quality of life of patients after resection of middle to low rectal cancer by avoiding damage to the pelvic nerves owing to better visualization of the dissection plane from the transanal access. Another important characteristic of every surgical procedure is the learning curve; in the case of TaTME, mastering the procedure requires a total of approximately 40–50 cases^30,31^. Notably, the study group included all patients treated with TaTME since its introduction at our center^19^. Thus, cases constituting the learning curve were also included in our study group, resulting in an additional negative impact on study outcomes.

The mean operative time in this study was 169 min. In the first literature reports regarding TaTME procedures, the surgery was performed by the same surgical team first from the abdomen and then from the transanal access, which significantly increased the total operating times^16,17,32^. At our center, each TaTME procedure had from the very beginning been performed simultaneously by two teams of experienced laparoscopists^19^.

Neoadjuvant oncological treatment (radiotherapy or chemoradiotherapy) plays a crucial role in management of rectal cancer^1–3^. According to current literature neoadjuvant therapy decreases local recurrence rates and increases resectability of tumors^1–5^. There is no need for neoadjuvant treatment in case of early stage of rectal cancer before surgery^1–5^. Nevertheless, in more advanced cases the neoadjuvant therapy is required^1–5,7^. In our study oncological treatment before operation was not required in 35 (27.34%) patients due to early stage of rectal cancer. In all other cases- 93 (72.66%) patients- neoadjuvant cancer therapy was used. Despite evidences stating efficiency of neoadjuvant treatment the matter of interval between neoadjuvant treatment and surgical treatment remains controversial, especially in case of radiotherapy and its radiation-induced toxicity. Nowadays, in primary rectal cancer, the interval after the end of the neoadjuvant treatment is 1 week in short-course of radiotherapy or 4–6 weeks in long-course of radiotherapy or chemoradiotherapy^1–5,7^. In our study the mean interval between the completion of neoadjuvant treatment and surgery was 11.87 (5–61) days, because most of the patients underwent short-course of radiotherapy. Radiotherapy improves oncological outcomes, but has negative influence on perioperative outcomes, because it worsens conditions during surgery^1–3^. That is why finding balance between perioperative outcomes and oncological outcomes is very important and TaTME procedure seems to be the best choice. TaTME helps to locate surgical planes to preserve the sphincter in patients after neoadjuvant treatment.

In a controversial study published in 2020, Wasmuth et al.^33^ reported the results obtained in 157 patients with rectal tumors operated on using the TaTME technique at seven hospitals in Norway between 2014 and 2018. Based on the results presented in this paper, the authors concluded that TaTME was associated with high complication and local tumor recurrence rates, with most recurrences having an unfavorable prognosis due to their extent or multifocal nature^33^. The study, presenting results contradictory to those published herein, has sparked a discussion about the usefulness of the TaTME technique in the treatment of rectal tumors^34^. Wasmuth et al.^33^ confirmed that TaTME is associated with a long learning curve, with the plateau being reached in virtually none of the Norwegian hospitals participating in the study. The relatively high rate of intraoperative complications reported in that study, including seven rectal perforations, two urethral injuries, and one bladder injury, reflects the technical errors made during the earliest phase of the TaTME learning curve^34^. Wasmuth et al.^33^ demonstrated that TaTME procedures should be performed at expert centers with extensive experience in laparoscopic colorectal surgery. Only at such centers, is it possible for a sufficiently large number of patients with middle and low rectal tumors to be reached and maintained to overcome the learning curve, thereby minimizing the risk of postoperative complications and optimizing the outcomes of oncological treatment. With growing experience, the acquisition of proper surgical techniques is possible at these expert centers to reduce the risk of local cancer recurrence.

Opposed to Wasmuth et al.^33^, in our study we present results from a single expert center that come from a large group of patients.

In addition to improved oncological outcomes, an indisputable advantage of the TaTME technique in the treatment of patients with rectal tumors is the preservation of sphincter function, which means that the quality of life of patients after surgical treatment of rectal cancer remains high, in contrast to open procedures involving the establishment of life-long colostomies, which are still frequently used in patients with low rectal tumors. Studies on the quality of life of patients after TaTME showed that functional outcomes after surgery were similar to those before surgery^35^. In addition, a similar incidence of low anterior resection syndrome (LARS)^36^ was demonstrated after LaTME and TaTME^37^. Although no detailed assessments were made regarding quality of life after TaTME in our study, most patients presented with LARS symptoms at consecutive outpatient follow-up visits. Notably, a temporary loop ileostomy was established in most of the patients in our study. Despite experiencing the symptoms of LARS after the ileostomy closure, none of the patients having had the experience of living with a temporary ileostomy would opt for a permanent stoma, which additionally confirms the plausibility of using the TaTME technique in the treatment of rectal cancer.

Five patients in our study group had rectal tumor in stage T4. In all T4 cases the tumor infiltrated prostate gland, which means that it invaded the surrounding organs in the lower rectum. In our opinion, the TaTME procedure is feasible for locally advanced tumors, but is large technical challenge for the operator and in these cases should be performed in expert centers only. In these cases there is high risk of incomplete resection and local recurrence. In our study R1 resection of the mesorectum was confirmed in 2 patients with T4 stage.

The main limitations of the study included a lack of randomization and the fact that the study was conducted in a selected group of patients from a single center.

This study showed that the TaTME procedure is an effective and safe method for the minimally invasive treatment of middle and low rectal cancers, particularly within an expert center setting. The better quality of dissection, owing to the more accurate visualization of the surgical field from the transrectal access during the TaTME procedure, facilitates complete removal of the mesorectum with adequate circumferential, proximal, and distal margins, directly translating to good oncological outcomes in short-term follow-up. No high rates of local recurrence were observed in the long-term follow-up, and the quality of life after the procedure was very good owing to preserved sphincter function.

## Data Availability

The datasets used and analyzed during the current study available from the corresponding author (matjagiel@gmail.com) on reasonable request.
